# Objective Auricular Measurements and Patient Satisfaction Following Cartilage-Cutting Otoplasty Using Fascioperichondrial Flaps

**DOI:** 10.3390/jcm15103795

**Published:** 2026-05-14

**Authors:** Timea Madaras, Mara Bărăbancia-Romete, Ruxandra Bodog, Horațiu-Paul Domnariu, Klara Brânzaniuc, Leonard Azamfirei, Bogdan-Andrei Suciu, Cristian Trâmbițaș

**Affiliations:** 1 Doctoral School of Medicine and Pharmacy, George Emil Palade University of Medicine, Pharmacy, Science and Technology of Targu Mures, 540139 Targu Mures, Romania; timea.madaras@umfst.ro (T.M.); klara.branzaniuc@umfst.ro (K.B.); leonard.azamfirei@umfst.ro (L.A.); bogdan.suciu@umfst.ro (B.-A.S.); 2 Department of Plastic and Reconstructive Surgery, Emergency Clinical County Hospital Targu Mures, 540139 Targu Mures, Romania; 3 Department of Surgery, University of Medicine and Pharmacy of Oradea, 410073 Oradea, Romania; bodogruxandra@gmail.com; 4 Surgical Clinical Department, Faculty of Medicine, Lucian Blaga University of Sibiu, 550024 Sibiu, Romania; horatiupaul.domnariu@ulbsibiu.ro

**Keywords:** cartilage-cutting otoplasty, fascioperichondrial flap, satisfaction

## Abstract

**Background/Objectives**: Prominent ears, affecting approximately 5% of the population, can cause significant psychosocial distress without functional impairment. **Methods**: This retrospective single-center study evaluated a cartilage-cutting otoplasty technique augmented by three triangular fascioperichondrial flaps in 40 patients (80 ears), aged 6–37 years (mean age: 13 years), treated between 2022 and 2024 at Târgu Mureș County Emergency Hospital. **Results**: Preoperative helical–mastoid distances (HMD) were elevated, superior: 21 mm, middle: 22 mm, inferior: 21 mm; concho-mastoid angle (CMA) averaged ~50°. Surgery involved postauricular skin excision, selective conchal cartilage resection, and flap fixation to the mastoid periosteum for stabilization. Measurements at immediate postoperative and 6-week follow-up showed significant reductions: superior HMD by 10 mm (immediate) and 9 mm (6 weeks), middle by 9 mm and 8 mm, inferior by 4 mm and 4 mm, CMA by 27° and 26° (all *p* < 0.001). Stability between postoperative and 6-week intervals was confirmed by ANOVA (no significant differences). Patient satisfaction was excellent: 75% very satisfied and 22% satisfied (97% overall positive), with no correlation with age or sex. **Conclusions**: This hybrid approach offers reliable correction, natural contours, and low postoperative complications, representing a feasible option in otoplasty. Longer-term prospective trials are recommended.

## 1. Introduction

Prominent ears represent one of the most frequent minor congenital deformities of the auricle, with an estimated prevalence of around 5% in the general population and a significant psychosocial impact despite the absence of functional deficit. Children with prominent ears are frequently subjected to teasing and stigmatization, which can lead to reduced self-esteem, social withdrawal, and long-term emotional consequences, while adults often perceive the deformity as a persistent disharmony of facial proportions. More than 200 otoplasty techniques have been described; cartilage-cutting methods, such as the Chongchet and Stenström techniques [1], reshape the auricle effectively but carry risks such as hematoma, skin necrosis, contour irregularities, and elevated recurrence rates [2]. In contrast, cartilage-sparing suture-based approaches, including the Mustardé and Furnas techniques, maintain tissue integrity, create more natural antihelical folds, and minimize cartilage trauma [3,4]. However, these methods still face issues like suture extrusion, granulomas, and recurrence in up to 25% of cases. Supportive strategies, notably postauricular fascial flaps and variants (e.g., perichondrio-adipo-dermal and modified bilateral fascioperichondrial flaps), counter these problems by overlaying a vascularized barrier along suture lines to reduce recurrence and complications [5].

The main purpose of the study was to evaluate the effectiveness and safety of a cartilage-cutting otoplasty technique combined with three triangular fascioperichondrial flaps in the correction of prominent ears, reviewing the anatomical and subjective results. Specific objectives included characterization of the study cohort; description of pre- and postoperative auricular measurements (helical–mastoid distance and concho-mastoid angle); analysis of early postoperative evolution at 6 weeks; and assessment of patient satisfaction using the Likert scale (very satisfied, satisfied, neutral, and dissatisfied) [6]. Secondary aims were to explore potential correlations between anthropometric changes and demographic variables (age, sex), as well as between satisfaction scores and objective parameters, in order to better define indications and prognostic factors for this technique. Furthermore, the study aimed to develop a follow-up protocol based on measurements of the ear that allow objective assessment of the otoplasty technique. We hypothesized that techniques using multiple fascioperichondrial flaps show optimal stabilization of the anthropometric measurements at 6 weeks postoperatively.

## 2. Materials and Methods

**Figure 1:**
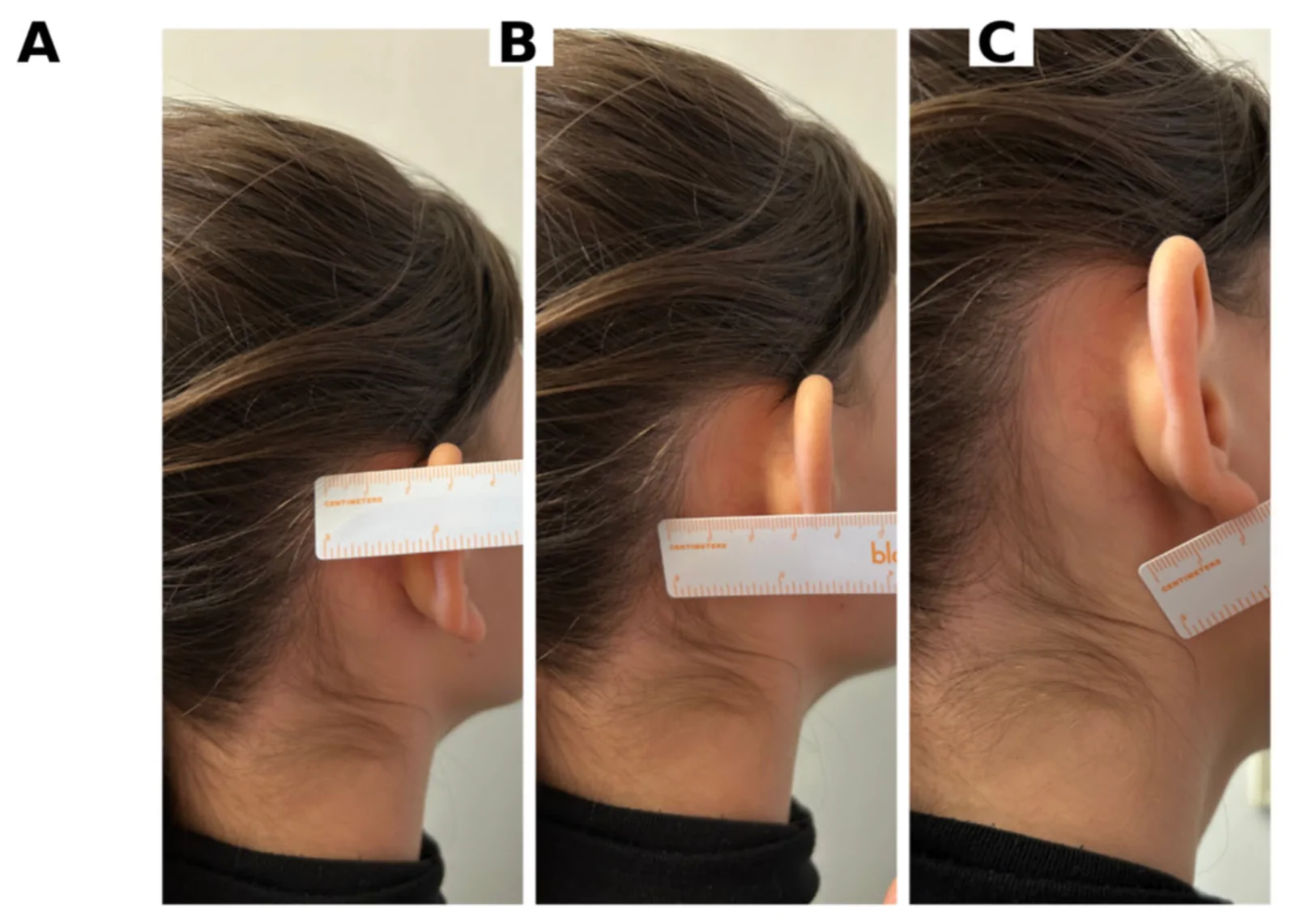
Clinical anthropometric measurement of bilateral auricular dimensions using a millimeter ruler. The photograph depicts a posterior-lateral view of a female patient’s ears. (**A**): superior HMD: Maximum vertical distance measured at the uppermost margin of the HMD region. (**B**): middle HMD: Vertical measurement taken at the central axis of the HMD region. (**C**):inferior HMD: Minimum vertical distance measured at the lowermost margin of the HMD region.

A retrospective, single-center study was conducted in the Plastic, Aesthetic, and Reconstructive Microsurgery Department of the County Clinical Emergency Hospital of Târgu Mureș over two years. Forty consecutive patients diagnosed with prominent ears who underwent bilateral otoplasty using the same cartilage-cutting technique combined with three triangular fascioperichondrial flaps were included, each ear being analyzed as an independent unit, for a total of 80 procedures. Inclusion criteria were as follows: signed informed consent (from patients or legal guardians), deformity corresponding to prominent ears without major associated auricular malformations, and surgery performed with the described technique; exclusion criteria comprised other surgical methods (e.g., cartilage-sparing techniques), operations performed in other institutions or by other specialties, additional congenital auricular anomalies, and incomplete clinical data. The study received a favorable opinion from the Medical Ethics Committee of the hospital (approval no. 26067/25 October 2024), allowing retrospective analysis of clinical records and discharge summaries. Objective evaluation was based on standardized anthropometric parameters: superior, middle, and inferior helical–mastoid distance (HMD) and concho-mastoid angle (CMA). Measurements were performed with the patient seated upright with the head in a natural position, using a flexible ruler and a goniometer, identifying precise anatomical landmarks on the helix (superior, middle, and lobular/inferior segments) and mastoid region. HMD superior was defined as the distance from the scalp to the upper helix, HMD middle as the distance at the mid-helix level, and HMD lobular/inferior as the distance from the base of the lobule to the scalp, while CMA represented the angle between the mastoid vertical line and the helix line [Figure 1]. McDowell’s widely cited study set optimal helix-to-mastoid distances (HMD) at 10–12 mm superiorly, 16–18 mm in the middle third, and 20–22 mm inferiorly with reference values of approximately 1–1.2 cm (superior HMD), 1.6–1.8 cm (middle HMD), 2.0–2.2 cm (inferior HMD) and 20–30° for CMA in esthetically normal ears [7]. Measurements were recorded preoperatively, immediately postoperatively, and at 6 weeks, and supplemented by standardized photographic documentation from frontal, oblique, lateral, and posterior views.

**Figure 2:**
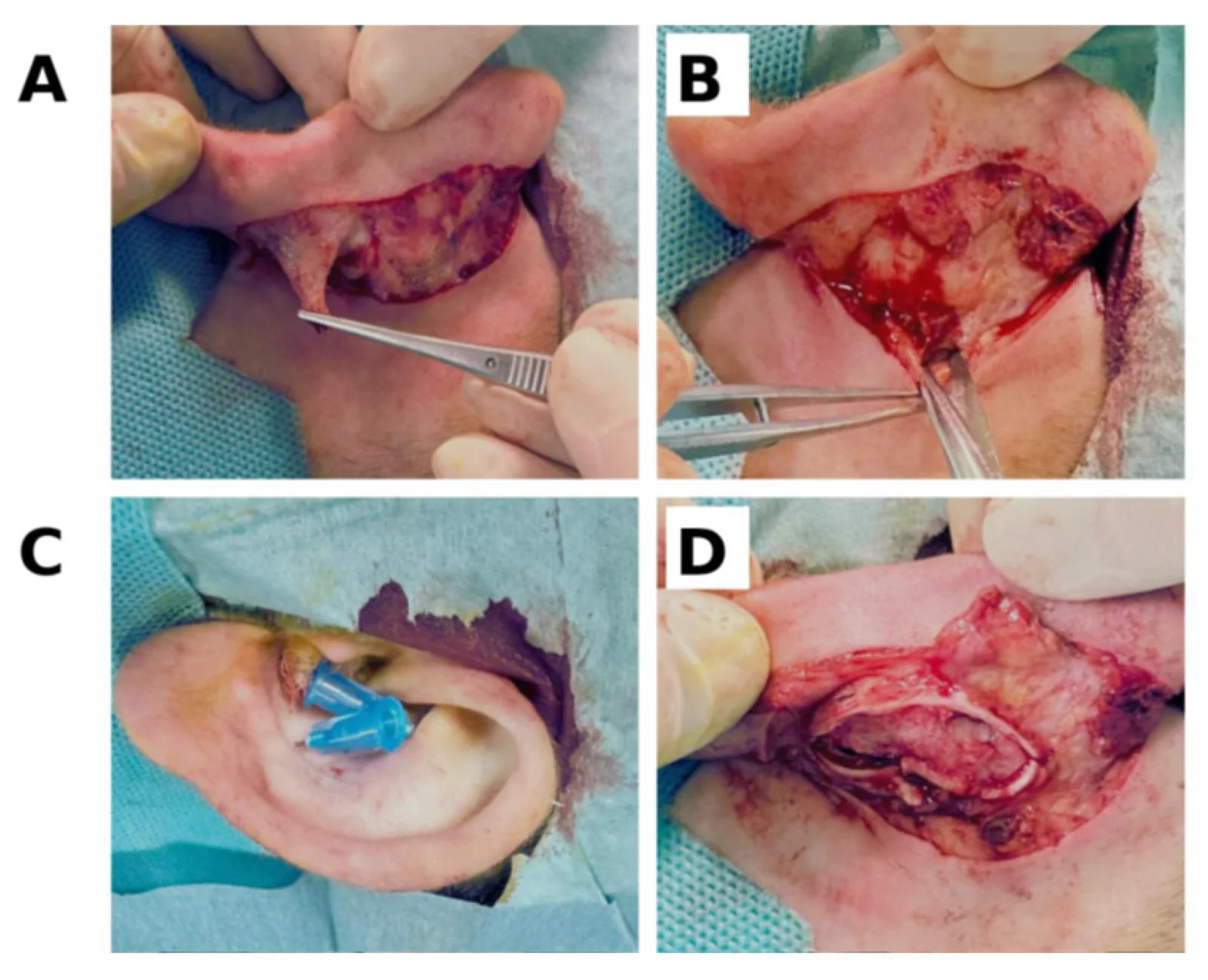
Intraoperative status, reviewing the main steps of the intervention: the creation of the fascioperichondrial flaps (**A**); making their fixation point at the level of the mastoid periosteum (**B**); anterior markings for the cartilage excision (**C**); excision of the auricular cartilage (**D**).

As for the surgical intervention, following antisepsis with povidone-iodine and sterile draping, an elliptical skin island was delineated in the postauricular region, positioned 3–4 mm from the postauricular sulcus and 5–6 mm from the helical rim. The subperichondrial plane was subsequently hydrodissected via injection of an anesthetic solution comprising lidocaine with epinephrine (1:200,000), followed by the excision of the retroauricular skin in a superficial plane to preserve deeper structures. Subsequent incision of the underlying fascia facilitated the creation of three triangular fascioperichondrial flaps. Meticulous subperichondrial dissection exposed the posterior auricular cartilage surface, which was precisely demarcated from the anterior aspect using 27-gauge needles to define the resection boundaries. The predetermined conchal cartilage segment was excised while rigorously preserving the anterior auricular skin; residual cartilage margins were then reapproximated with nonabsorbable monofilament sutures (3-0, 4-0) elected based on patient age and cartilage rigidity. These sutures were strategically placed to avert anterior skin perforation and to reestablish an esthetically balanced auriculomastoid relationship, avoiding hypercorrection. The fascioperichondrial flaps were mobilized anteriorly and anchored to the mastoid periosteum, thereby furnishing robust posterior stabilization. Retroauricular skin closure was completed with continuous 4-0 monofilament sutures [Figure 2].

Following surgical completion, an atraumatic dressing was applied over the suture line, augmented with gauze padding and a compressive head bandage. Sutures were removed between postoperative days 10 and 14, at which point the bulky bandage was discontinued; patients were then instructed to wear a headband continuously during daytime hours for 4 weeks and nocturnally for an additional 2–4 weeks. Showering was permitted after the initial dressing change, whereas swimming was prohibited during the first postoperative month to eliminate the risk of infection. Patient satisfaction was evaluated using the Likert scale, with scores ranging from very satisfied to dissatisfied. Data were analyzed using the Posit team (2025). RStudioDesktop (version 2026.04.0+526): Integrated Development Environment for R tool. Continuous variables were expressed as means ± standard deviations, while categorical variables were summarized as absolute numbers.

## 3. Results

The study included 40 patients (80 ears) operated using the same otoplasty technique; 27 (67%) were male and 13 (33%) were female. Age ranged from 6 to 37 years, with a mean age of 13 years and a range of 31 years; most patients were under 10 years, which the authors considered an optimal window for correction. The age distribution was asymmetric and showed a bimodal pattern, with one cluster around 10–20 years and a smaller group over 30 years, reflecting a tendency to seek correction predominantly in childhood and adolescence.

Preoperatively, the mean superior HMD was 21 mm, the middle HMD 22 mm, and the inferior HMD 21 mm. These values exceeded the typical esthetic reference values, confirming a significant ear–scalp projection in all three segments of the helix. We followed our patients postoperatively and analyzed the same parameters immediately postoperatively and at a 6-week follow-up. [Table 1].

**Figure 3:**
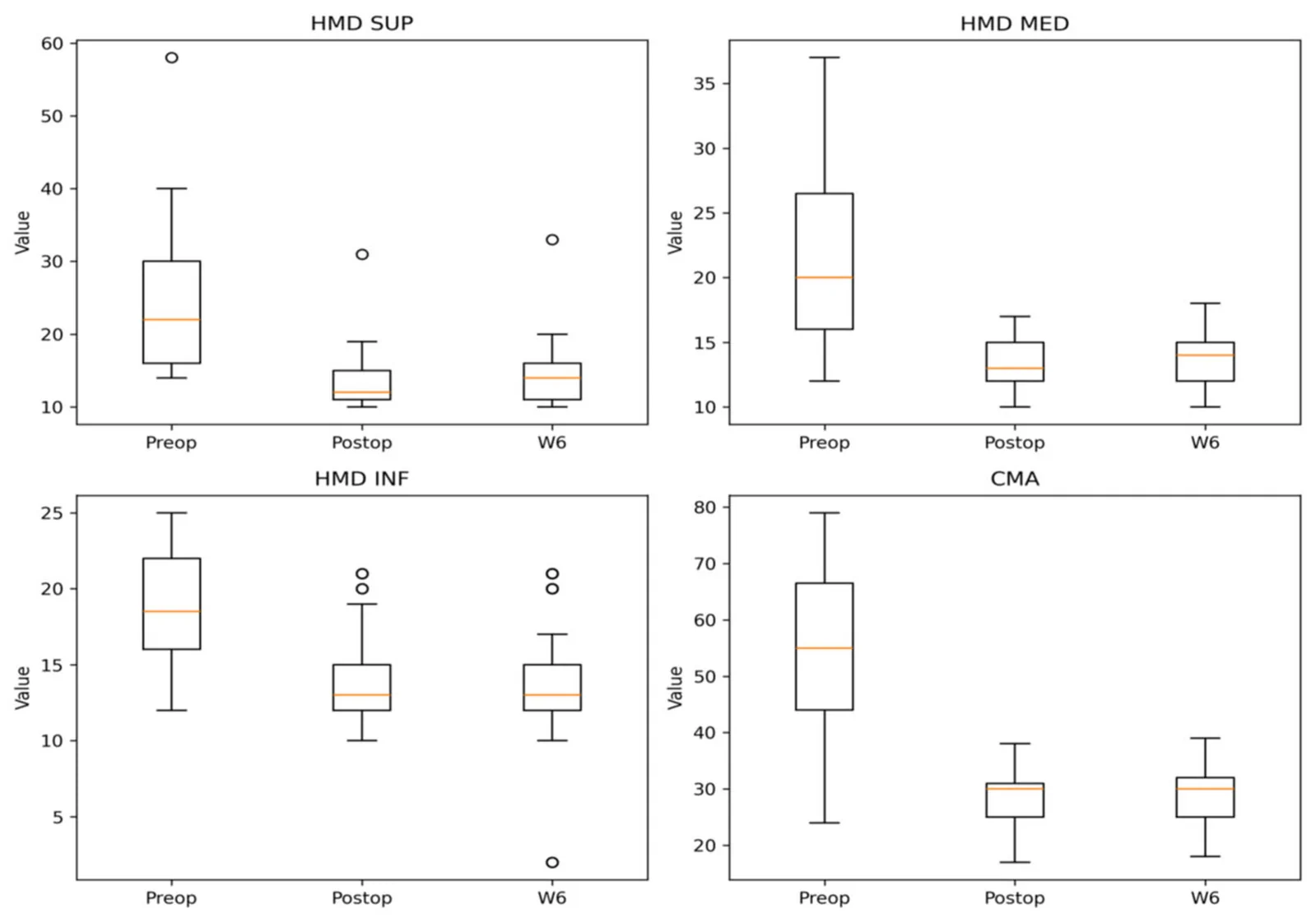
Box-and-whisker plots of pre- and post-intervention volume measurements across anatomical regions using paired *t*-tests. Paired comparisons (Pre vs. Post) revealed comparable distributions in most panels, with modest Post reductions observed in INF and CMA subgroups.

Our surgical intervention resulted in statistically and clinically significant reductions across all evaluated parameters (HMD SUP, HMD MED, HMD INF, and CMA). The therapeutic effect demonstrated consistency throughout the patient cohort, substantiated by high t-test values and narrow confidence intervals. Paired t-tests demonstrated a statistically significant reduction in all measured parameters following surgery. The superior helical–mastoid distance (HMD SUP) decreased by a mean of 10 mm (95%, *p* < 0.001), while the middle (HMD MED) and inferior (HMD INF) distances decreased by 9 mm (95%, *p* < 0.001) and 4 mm (95%, *p* < 0.001), respectively. The concho-mastoid angle (CMA) showed the largest change, with a mean reduction of 27° (95%, *p* < 0.001) [Figure 3]. These findings support the efficacy of the surgical procedure in correcting auricular prominence. Paired t-tests demonstrated statistically significant reductions in all evaluated parameters between preoperative and 6-week postoperative measurements. The superior helical–mastoid distance decreased by a mean of 9 mm (95%, *p* < 0.001), while the middle and inferior distances decreased by 8 mm (95%, *p* < 0.001) and 4 mm (95%, *p* < 0.001), respectively. The concho-mastoid angle showed a substantial and persistent reduction of 26° (95%, *p* < 0.001).

**Figure 4:**
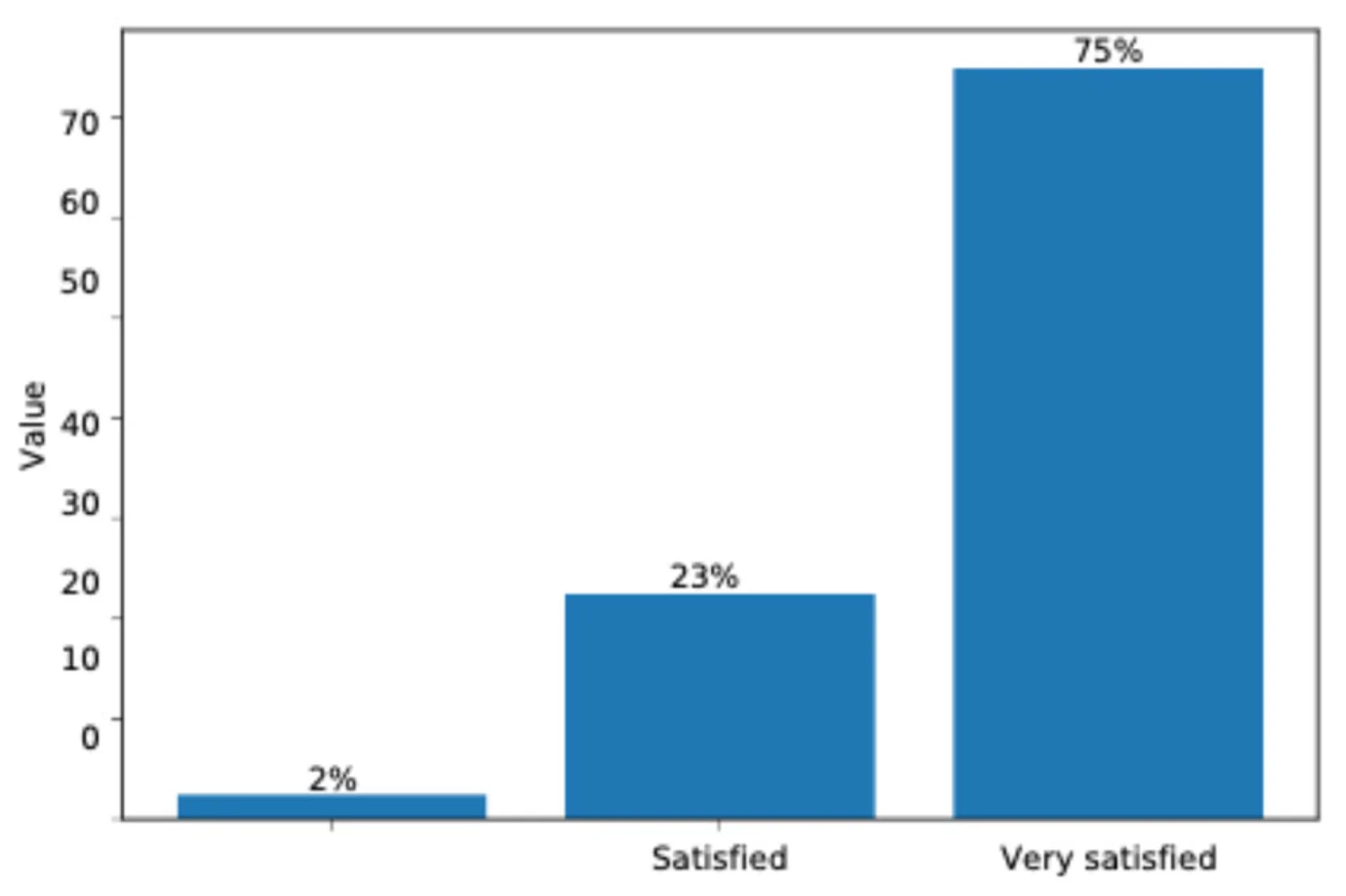
Distribution of patient satisfaction levels following intervention. Bar graph illustrates the percentage of patients reporting neutral (2%), satisfied (23%), and very satisfied (75%) outcomes, as measured on a categorical satisfaction scale.

Repeated measures ANOVA revealed a significant effect for all evaluated parameters. Post hoc paired comparisons demonstrated significant reductions between preoperative and postoperative measurements, as well as between preoperative and 6-week measurements, while no significant differences were observed between postoperative and 6-week values, indicating stability of the surgical outcome. Mean values of HMD SUP, HMD MED, HMD INF, and CMA at preoperative, immediate postoperative, and 6-week follow-up time points. A significant reduction was observed following surgery, with stable values between postoperative and 6-week measurements (Figure 4).

**Figure 5:**
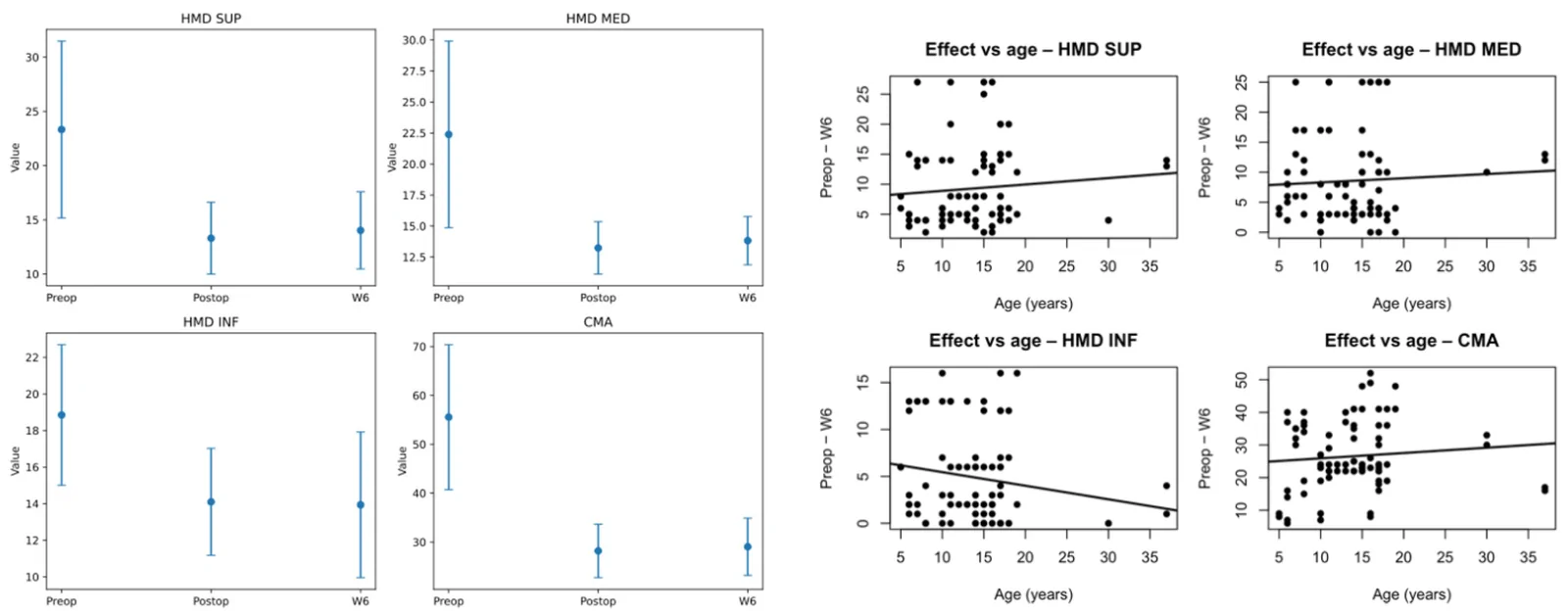
The (**left**) panel shows bar graphs with error bars for ANOVA analysis, comparing pre-intervention (Pre), post-intervention (Post), and week 6 follow-up (W6) values across the same subgroups, highlighting temporal changes and statistical group differences typical in longitudinal clinical trials. The (**right**) panel displays four scatter plots with regression lines illustrating the relationship between effect size and patient age (*x*-axis, years) for HMD-defined superior (SUP), medial (MED), and inferior (INF) regions, plus CMA, where data points represent individual outcomes and trends indicate age-related variations in treatment response.

Scatter plots revealed no clinically relevant association between patient age and the magnitude of surgical correction maintained at 6 weeks for any of the evaluated parameters [Figure 5]. Linear regression analysis demonstrated minimal slopes, indicating that the surgical outcome was largely independent of patient age.

Patient satisfaction was assessed in person at the 6-week postoperative follow-up using a standardized questionnaire based on a 4-point Likert scale (very satisfied, satisfied, neutral, and dissatisfied). Satisfaction levels were high, with 75% of patients reporting being very satisfied and 23% satisfied, while 2% reported neutral satisfaction and no patients reported dissatisfaction [Figure 4]. Overall, 97% of patients were satisfied or very satisfied, reflecting a high level of acceptance of the surgical outcome. Early postoperative complications were systematically evaluated at the same follow-up visit using predefined clinical criteria and included hematoma, infection, skin necrosis, and suture-related complications; none of these events were observed in the study cohort.

## 4. Discussion

The present analysis demonstrates that otoplasty using three fascioperichondrial flaps produces both statistically and clinically significant improvements in auricular positioning across all evaluated parameters, with good outcomes and low complication rates maintained through the 6-week postoperative follow-up period [8]. The consistent reduction in helical–mastoid distance measurements at both immediate postoperative and 6-week intervals confirms the sustained effectiveness of the surgical intervention and a favorable long-term outcome. The magnitude of change was the greatest in the superior and middle thirds of the auricle, consistent with the clinical importance of these regions for frontal and oblique facial views, while the inferior segment (lobule) remained within a natural protrusion range, thus avoiding a “stuck-on” appearance [9,10]. The superior helical–mastoid distance reduction of 10 mm immediately postoperatively [Figure 5], with a preservation of 9 mm at six weeks, reflects the reliability of the surgical technique in achieving lasting auricular correction [11,12]. The present technique, based on selective cartilage excision and posterior fascioperichondrial flap fixation, provides a stable reduction in conchal projection while preserving a smooth and natural anterior contour, which is particularly advantageous in severe deformities and in older patients with stiffer cartilage [13].

The concho-mastoid angle measurements, which demonstrated the most substantial changes (mean reduction of 27° immediately postoperatively and 26° at six weeks), align with contemporary surgical standards emphasizing correction of the conchal component as a critical feature in prominent ear deformities. These measurements fall within the established esthetic reference ranges of 10–12 mm in the upper third, 16–18 mm in the middle third, and 20–22 mm in the lower third, documented in previous landmark studies [14]. The minimal loss of correction between immediate postoperative and six-week measurements suggests excellent mechanical stability of the surgical reconstruction in a short-term follow-up.

The narrow confidence intervals and high t-test values reported across all parameters provide robust statistical evidence of surgical efficacy. The achievement of *p* < 0.001 for all primary outcome measures demonstrates not only statistical significance, but also substantial clinical effect sizes. This finding has important clinical implications regarding the timing of postoperative assessment and patient counseling. Previous studies have documented that most patients experience significant improvement in swelling within 2–3 weeks, with mild residual edema persisting through week 6. The stability of measurements between the postoperative period and the six-week follow-up likely reflects true surgical and clinical outcomes, rather than the effect of the fluctuating edema [15,16], which resolves in the first three weeks of the postoperative period.

**Figure 6:**
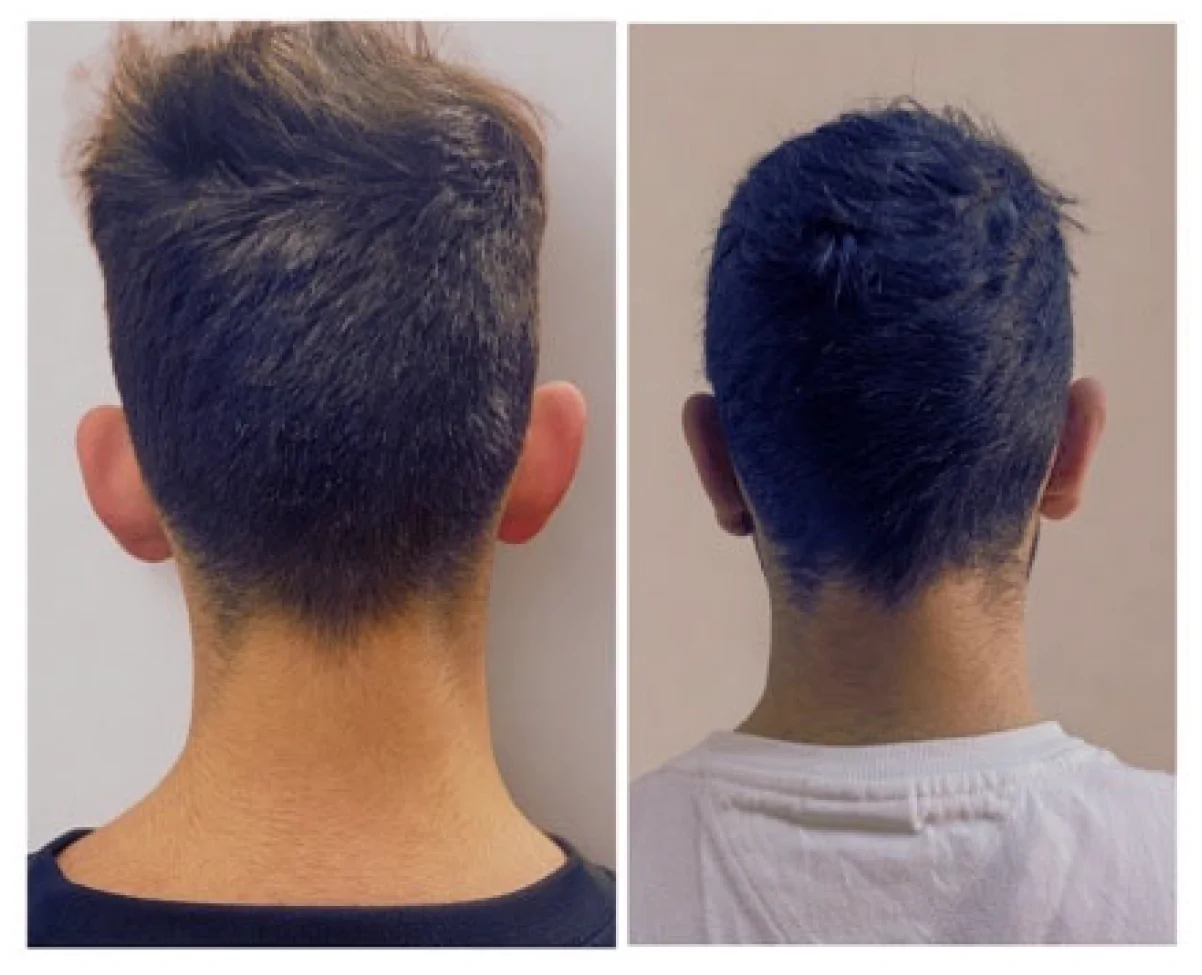
Preoperative and postoperative posterior views demonstrating clinical outcomes.

**Figure 7:**
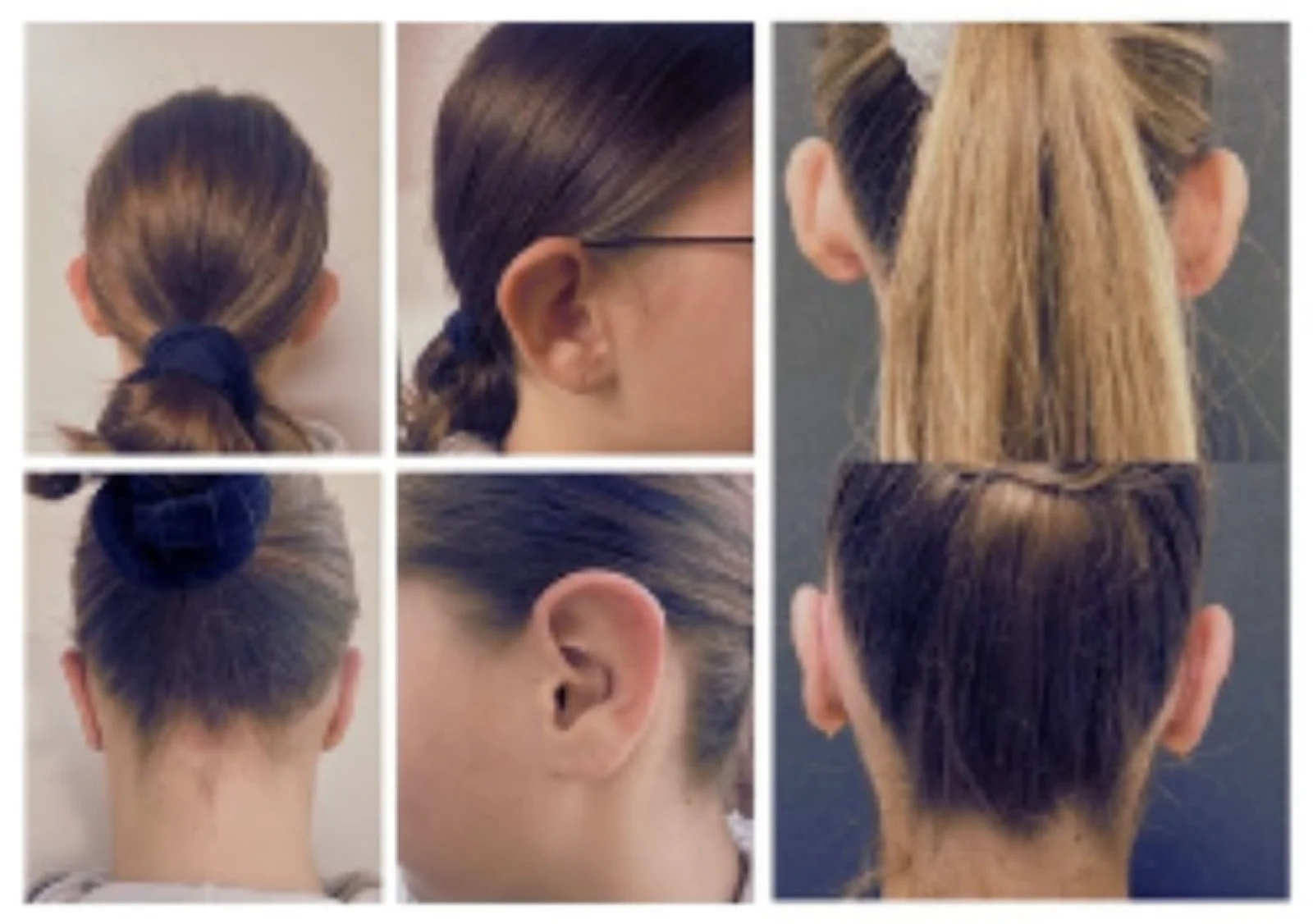
Serial multi-view photographs documenting otoplasty progression.

The results of this study are consistent with contemporary publications evaluating combined otoplasty techniques. While the current study does not report recurrence rates, the stability of measurements through six weeks and the absence of significant differences between immediate postoperative and six-week measurements suggest favorable conditions for long-term durability. The application of multiple corrections addressing both antihelix formation and concho-mastoid positioning likely contributes to this stability [Figure 6 and Figure 7].

The bimodal age distribution observed in this cohort, with predominant clustering in the 10–20-year age group and a secondary group over 30 years, reflects established patterns in otoplasty case selection and patient motivation for surgical correction [17,18]. In our surgical interventions, increased auricular cartilage rigidity was taken into consideration, and multiple technical modifications were employed to facilitate adequate reshaping. These included slightly increased suture tension and, in selected cases, the use of additional Mustardé sutures to achieve stable fold definition. Furthermore, limited conchal cartilage reduction was performed to further decrease auriculocephalic projection. These adjustments allowed for consistent esthetic outcomes despite the greater stiffness typically encountered in this age group. The surgical outcome was largely independent of patient age, as demonstrated by minimal slopes in linear regression analysis, which provides valuable evidence regarding cartilage plasticity across the developmental spectrum. While pediatric patients with more malleable cartilage may theoretically require less aggressive surgical techniques, and adults with stiffer cartilage may necessitate more invasive approaches involving cartilage scoring or excision, the current study suggests that appropriately selected surgical techniques can achieve comparable correction regardless of age [19].

This age-independent efficacy contrasts with some literature, suggesting that adult cartilage rigidity presents technical challenges that may compromise long-term stability. However, recent advances in combination techniques employing both antihelix reshaping (through Mustardé-type sutures) and concho-mastoid fixation (through Furnas-type sutures) have substantially improved outcomes across age groups [20]. The current cohort’s consistent therapeutic effect across the age range indicates that appropriate case selection and comprehensive surgical planning addressing multiple auricular components, rather than relying on single-technique approaches, yield superior and reproducible results.

The predominance of patients under 20 years in this series suggests that early intervention may prevent the psychological sequelae associated with prominent ear deformity during critical developmental and social periods. The timing of surgical correction before or during early adolescence aligns with recommendations from multiple authors emphasizing the psychological importance of addressing the deformity during this vulnerable developmental window [21,22].

Although the present work focuses primarily on objective measurements and satisfaction, it is framed against the known spectrum of otoplasty complications, whose reported early incidence ranges from 0% to 8.4%, with late complications varying widely up to 47.3% in some series [23,24]. The combination of careful preoperative planning, atraumatic dissection in the subperichondrial plane, and secure fixation of the fascioperichondrial flaps to the mastoid periosteum is designed to reduce the risk of hematoma, perichondritis, scar hypertrophy, and contour deformity [25,26]. In addition, the standardized postoperative regimen with compressive bandaging and prolonged headband use aims to protect the corrected auricular position during the critical healing period.

The study has few limitations inherent to its retrospective, single-center design and relatively small sample size, which may limit the generalizability of the findings. The follow-up period analyzed in detail was limited to 6 weeks; although this interval allows assessment of early stability, it does not capture potential late recurrences, scar maturation, or long-term patient satisfaction. Furthermore, the absence of a comparative control group treated with alternative techniques (e.g., Mustardé/Furnas or cartilage-sparing otoplasty) precludes direct conclusions on the superiority of the studied method versus other established approaches. This study design includes 40 patients, with both ears analyzed, resulting in 80 evaluated units. While each ear was considered separately to allow for a more detailed assessment of surgical outcomes, it is recognized that bilateral observations within the same patient are not entirely independent and may introduce intra-subject correlation. This methodological aspect could influence variance estimates and statistical significance. Nevertheless, this approach is frequently adopted in otoplasty research and remains appropriate for capturing ear-specific outcomes. The findings should therefore be interpreted in this context, and future studies applying statistical models that account for within-patient correlation, such as mixed-effects analyses, may further refine the robustness of these results.

## 5. Conclusions

Cartilage-cutting otoplasty combined with three triangular fascioperichondrial flaps proved to be an effective and stable technique for the correction of prominent ears, bringing HMD and CMA values into the accepted esthetic ranges and maintaining these corrections at least up to six weeks postoperatively. The procedure was associated with very high patient satisfaction, independent of age, sex, or environment of origin, supporting its role as a reliable option in the surgical armamentarium for prominent ear deformity. The moderate influence of age on residual superior projection suggests that early intervention remains preferable, but satisfactory results can also be obtained in older patients when the technique is carefully individualized. Further prospective studies with larger cohorts, longer follow-up, and direct comparisons with cartilage-sparing and suture-only methods are warranted to more precisely define indications, refine technical details, and establish long-term outcome profiles for this approach.

## Figures and Tables

**Table 1:** Presentation of the resulting values on short- and long-term follow-up.

Parameter	Preoperative (Mean)	Immediate Postoperative (Mean)	6 Weeks Postoperative (Mean)	*p*-Value
HMD (Superior)	21 mm	13 mm	13 mm	<0.001
HMD (Middle)	22 mm	13 mm	13 mm	<0.001
HMD (Inferior)	21 mm	13 mm	13 mm	<0.001
CMA	55°	28°	29°	<0.001

## Data Availability

The original contributions presented in this study are included in the article. Further inquiries can be directed to the corresponding authors.
